# Endothelial follicle-stimulating hormone receptor expression in invasive breast cancer and vascular remodeling at tumor periphery

**DOI:** 10.1186/s13046-015-0128-7

**Published:** 2015-02-05

**Authors:** François Planeix, Mohammad-Ahsan Siraj, François-Clément Bidard, Blaise Robin, Christophe Pichon, Xavier Sastre-Garau, Martine Antoine, Nicolae Ghinea

**Affiliations:** INSERM “Tumoral Angiogenesis” Laboratory, Curie Institute, Research Center, Translational Research Department, 26 rue d’Ulm, Paris, France; Curie Hospital, Oncology Department, 26 rue d’Ulm, Paris, France; Curie Hospital, Pathology Department, 26 rue d’Ulm, Paris, France; Tenon Hospital, Pathology Department, 4 rue de la Chine, Paris, France

**Keywords:** Angiogenesis, Breast cancer subtype, CD34, Estrogen receptor, FSHR, HER2, Lymphangiogenesis, Podoplanin, Progesterone receptor, Triple negative breast cancer, Vascular remodeling

## Abstract

**Background:**

Follicle-stimulating hormone receptor (FSHR) is expressed on the endothelial surface of blood vessels associated with solid tumor periphery, where angiogenesis is known to occur. The correlation between FSHR expression and formation of new peritumoral vessels has not been previously investigated.

**Methods:**

We used immunohistochemical techniques involving specific antibodies to detect FSHR and the endothelial markers (CD34, VEGFR2, and D2-40) in tissue samples from 83 patients with lymph node-negative, invasive breast cancer representing four main clinical treatment groups: HR+/HER2-, HR+/HER2+, HR-/HER2+ and triple-negative.

**Results:**

The FSHR+ vessels were exclusively located at breast cancer periphery, in a layer that extended 2 mm into and 5 mm outside of the tumor. The percentage of blood vessels expressing FSHR reached a maximum of 100% at the demarcation line between the tumor and the normal tissue. Common among FSHR+ vessels, regardless of breast cancer type, were the high densities of arterioles and venules (6.4 ± 1.4 and 13.9 ± 2.1 vessels/mm^2^, respectively). These values were 3-fold higher that those noticed for CD34+ arterioles and venules associated with normal breast tissue located at a distance greater than 10 mm outside the tumors. The average density of FSHR+ and CD34+ blood vessels as well as of D2-40+ lymphatic vessels did not differ significantly among breast cancer subgroups. FSHR+ vessels did not express VEGFR2. The endothelial FSHR expression correlated significantly with the peritumoral CD34+ vessels’ density (p < 0.001) and tumor size (p = 0.01).

**Conclusion:**

Endothelial FSHR expression in breast cancer is associated with vascular remodeling at tumor periphery.

## Background

Breast cancer was the leading cancer type in women in all countries of Europe in 2012. The overall estimate of 464,000 new cases was the equivalent of about 1,268 new breast cancer diagnoses each day [1]. In Unites States the breast cancer was expected to account for 28.7% (232,670) of all new cancer cases among females in 2014 [2].

Breast cancer is a heterogeneous disease with varied morphological appearances, molecular features, behavior and response to therapy. Based on the expression of estrogen and progesterone hormone receptors (ER and PR, respectively) and human epidermal growth factor receptor 2 (HER2), breast cancers can be classified into four major clinical treatment groups: (i) tumors positive for ER, PR or both but negative for HER2, (ii) tumors positive for ER, PR or both and positive for HER2, (iii) tumors HER2-positive (positive for HER2 but negative for ER and PR) and (iv) triple-negative (TN) (negative for ER, PR and HER2) [3].

Hormone receptor status and HER2 overexpression have been identified as important predictors of patient outcome, including risk of locoregional recurrence, distant metastasis, and survival in breast cancer patients. Approximately 66% of breast cancers are hormone receptor (HR) positive tumors. Overexpressed in 15%-20% of human breast cancers [4], HER2 is associated with increased risk of locoregional recurrence [5] and increased breast cancer mortality [6]. TN represents 15-20% of all breast cancers [7]. It is a highly aggressive with higher rates of relapse and shorter overall survival in the metastatic setting compared with other subtypes of breast cancer. To date, not a single targeted therapy has been approved for the treatment of TN, and cytotoxic chemotherapy remains the standard treatment [8].

Analysis of genomic and proteomic expression profiles of oncogenic signaling pathways have established various intrinsic subtypes of breast cancer (luminal A, luminal B, HER2-enriched, basal-like, claudin-low) and a normal breast-like group [9]. These subgroups were associated with different disease outcomes, suggesting a biologic basis behind the clinical heterogeneity of breast cancer [8,10]. To improve patient outcomes, an integration of the intrinsic subtypes with the four main clinical treatment groups (HR+/HER2-, HR+/HER2+, HR-/HER2+ and triple-negative) has been envisaged [11].

Improved molecular understanding of breast cancer has resulted in identification of various cancer cell targets for diagnostic and therapeutic interventions. Unfortunately, tumor heterogeneity hampers tumor-specific targeting [12-14]. The problem of tumor heterogeneity is supposed to be reduced by targeting the tumor-associated vasculature. The latter, a ubiquitous component of cancer, is essential for tumor growth and metastasis [15]. Therefore, depriving a tumor from its oxygen and nutrients, either by preventing the formation of new vessels (angiogenesis), or by disrupting vessels already present in the core of tumors (“anti-vascular therapy”), appears to be an effective treatment modality in oncology [16-18]. However, the efficacy of these therapies is substantially compromised by the inability of drugs to completely kill tumor cells located at the periphery of the tumor mass [19]. Therefore, the future of antivascular cancer therapy may depend on finding new targets on peripheral and peritumoral vessels [20].

The presence of specific endothelial cell markers exposed on the luminal surface of tumor peripheral vessels may offer an opportunity for marker-specific delivery of drugs. We have obtained evidence that this is the case for the follicle-stimulating hormone receptor (FSHR), a G-protein linked receptor that binds FSH, a key hormone in mammalian reproduction. FSHR was shown to be expressed selectively on the luminal surface of tumor blood vessels. A general characteristic of the blood vessels that express the endothelial FSHR is that they are located at the periphery of the tumors [21-23], in shells that have a thickness of approximately 10 mm (range, 7 to 15) and extended a few millimeters both inside and outside the tumor in the apparently normal tissue. No FSHR-expressing vessels were detected in the deeper areas of the tumors.

The observation that in invasive breast cancers the endothelial cells proliferate at the tumor periphery, but not in the interior [24], suggested that FSHR expression by endothelial cells may be associated with their proliferation in this particular location. However, the correlation between FSHR expression and formation of new peritumoral blood vessels has not previously investigated. Hence, we analyzed the expression of FSHR and its correlation with markers of angiogenesis in 83 node-negative breast cancer samples representing four main clinical treatment groups: HR+/HER2- tumors, HR+/HER2+ tumors, HR-/HER2+ tumors, and TN tumors.

## Methods

### Patients and tissue specimens

The specimens were resected at the Curie Hospital, Paris (45 patients) and Tenon Hospital, Paris (38 patients). The median age of patients at time of diagnosis was 55 (range, 27–75 years). All breast cancer patients had node-negative invasive tumors and had not received any therapy before surgery. Receptor status for estrogen, progesterone, and HER2 was known for all patients. The tumors were graded according to the Scarff-Bloom-Richardson scoring system by two pathologists (MA and XS). Tissue specimens were fixed in formalin and embedded in paraffin.

This study was approved by the Curie Institute and Tenon hospital ethical boards. A waiver for signed informed consent was obtained due to the retrospective nature of the study.

### Antibodies

The FSHR-highly specific monoclonal antibody 323 was produced in ascites and purified as described [25]. Biotinylated goat anti-mouse IgG (Fc specific) and streptavidin-horseradish peroxidase conjugate were purchased from Sigma-Aldrich, Saint Quentin Fallavier, France. Mouse anti-human CD34 (clone QBEnd 10) and mouse anti-human podoplanin (clone D2-40) monoclonal antibodies were purchased from Dako A/S, Glostrup, Denmark. Rabbit monoclonal anti-HER2/neu (clone 4B5), rabbit monoclonal anti-ER (sp1), and rabbit monoclonal anti-PR (IE2) were from Ventana Medical Systems Inc., Oro Valley, AZ, USA. Rabbit monoclonal antibody anti VEGFR2 (clone 55B11) was from Cell Signaling Technology, Danvers, MA, USA.

### Chemicals

Sodium borohydride, 3-amino-9-ethylcarbazole (AEC), sodium azide, hydrogen peroxide 30%, goat serum, and haematoxylin Gill solution n°3 were purchased from Sigma-Aldrich, Saint-Quentin Fallavier, France. Shandon Immu-Mount medium was obtained from Thermo-Scientific, Asniere sur Seine, France. Streptavidin/Biotin blocking kit was purchased from Vector laboratories, Burlingame, USA.

### Immunohistochemistry

Serial 5-μm-thick sections were cut, attached to SuperFrost slides, deparaffinized with xylene, dehydrated gradually in ethanol and washed with running tap water for 60 min. Access to tissue antigen sites for antibody attachment was enhanced by incubating slides at 90°C for 40 min with 10 mM citrate buffer, pH 6. After cooling at room temperature (RT) for 20 min, and after each subsequent step, slides were rinsed with PBS. To block endogenous peroxidase activity, the sections were incubated with 6% hydrogen peroxide (15 min at RT). Sodium borohydride (10 mg/ml PBS) was used to quench free aldehyde groups (15 min). Non-specific binding of antibodies was blocked by incubating slides with 2% goat serum in PBS (blocking buffer) at RT for 2 h. The slides were incubated with monoclonal primary antibody (5 μg/ml FSHR323) in blocking buffer overnight at 4°C. Goat anti-mouse IgG (Fc-specific) coupled to horseradish peroxidase (dilution 1:500) was used as secondary antibody. As chromogen we used AEC. Normal-appearing tissue located at a distance greater than 10 mm outside the tumors was used as control. The sections were washed in distilled water containing 0.1% sodium azide, counterstained with Gill’s haematoxylin for 10 s, and mounted in Shandon Immu-Mount medium. Immunohistochemistry for CD34 and D2-40 was carried out according to the Dako’s protocol.

### Immunofluorescent confocal microscopy

We used immunofluorescence confocal microscopy to co-localize FSHR with ER, PR, HER2, and VEGFR2 in tissue sections of breast cancer patients. For antigen retrieval we used 10 mM of citrate buffer, pH6. For colocalization experiments of FSHR with VEGFR2 the antigen retrieval buffer we used 10 mM Tris–HCl/1 mM EDTA, pH9. As primary antibodies we used mouse anti-human FSHR 323 monoclonal antibody (5 μg/ml), rabbit monoclonal anti-ER (dilution: 1 μg/ml), rabbit monoclonal anti-PR (dilution: 1 μg/ml), rabbit monoclonal anti-HER2/neu (dilution: 6 μg/ml), and rabbit monoclonal anti-human VEGFR2 antibody (dilution: 10 μg/ml). Goat anti-mouse IgG coupled to Alexa555 and goat anti-rabbit immunoglobulin coupled to Alexa 488 were used as secondary antibodies. The nuclei were detected by incubating slides with DAPI added to the secondary antibody mixtures (dilution 1:1,000). The slides were mounted in Dako fluorescent mounting medium containing 15 mM sodium azide and examined with a Zeiss 700 Confocal Laser Scanning Microscope. Negative controls consisted of breast samples incubated only with fluorescent secondary antibody mixtures.

### Statistical analysis

We analyzed the markers’ expression in a band with a width of 10 mm, centered on the demarcation line between the tumor and the normal breast tissue, on breast cancer tissue sections stained by standard peroxidase-immunohistochemistry. We have quantitatively determined the density of FSHR-, CD34-, D2-40-positive blood vessels. This was done by counting the number of marker-positive vessels on digital images of five “hotspots” per patient from whole images of serial sections obtained by using the Philips Digital Ultra-Fast Scanner 1.6 RA and Philips Image Management System 2.2RA, available in Curie Institute. Capillaries, arterioles, and venules were identified as previously described [26]. The percentage of the area occupied by CD34+ endothelial cells was calculated as previously described by [27] using the formula: % of endothelial cells area = Σ pixels of endothelial cells area × 100 / Σ pixels of the whole picture. The pixel density of the image was measured with the “colour deconvolution function” of FIJI software in the range of 0 – 255, where 0 means presence of endothelial cells and 255 means absence of endothelial cells. Values presented are means ± standard deviation (SD). Relationships between the vascular FSHR expression and CD34, D2-40, and clinicopathological data were examined by Pearson's correlation coefficient. Results were analyzed for statistical significance with 2-tailed probability values, with p < 0.05 considered significant.

## Results

### FSHR expression as function of molecular subtype of invasive breast cancer

**Figure 1:**
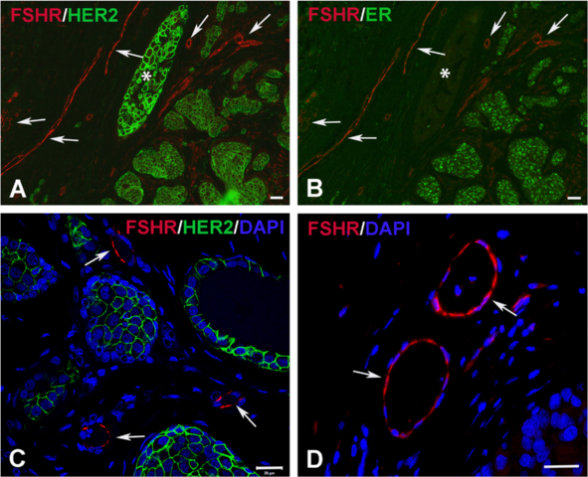
**Expression of FSHR in invasive breast cancer as function of molecular subtype of tumors.** Immunofluorescence confocal microscopy analysis was performed on paraffin-embedded sections of human breast cancer tissues with the use of the anti-FSHR monoclonal antibody 323, followed by a red-labeled secondary antibody. Irrespective of molecular subtype, breast tumors showed strong staining of blood vessels **(A through D)**. Anti-HER2 and anti-ER antibodies, followed by a green-labeled secondary antibody, confirmed the molecular subtype of breast tumors. Green signals from rabbit antibodies against HER2 **(A and C)** and estrogen receptor **(B)** were merged with the red signal from the anti-FSHR antibody. Sections were also stained with DAPI **(C and D)**. **(A,B)** HR+/HER+ breast tumors. Please note that tumor cells with strong signal for HER2 (asterisk) did not express the estrogen receptor. **C)** HR-/HER2+ tumors; **D)** triple negative tumors. The scale bar represents 20 μm in all panels.

We analyzed 83 breast cancer patients: 35 had HR+/HER2- tumors, 13 HR+/HER2+ tumors, 19 HR-/HER2+ tumors, and 16 had TN tumors (Table 1). All 83 patients examined expressed FSHR in endothelial cells. Representative immunofluorescence confocal microscopy pictures of FSHR expression as function of breast cancer molecular subtype are shown in Figure 1.

**Table 1:** **The expression of FSHR, CD34, and D2-40 in peritumoral vessels associated with invasive breast cancer**

		**Marker immunostained vessels/mm** ^**2 a**^			**p-value**		
**Tumors**	**No of cases**	**FSHR**	**CD34**	**D2-40**	**FSHR vs. CD34**	**FSHR vs. D2-40**	**CD34 vs. D2-40**
**HR+/HER2-**	**35**	**36.7 ± 4.0**	**100.4 ± 26**	**3.9 ± 3.7**	**p < 0.001**	**p > 0.05**	**p < 0.01**
**HR+/HER2+**	**13**	**31.4 ± 5.2**	**92.4 ± 13.9**	**5.4 ± 4.7**	**p < 0.001**	**p > 0.05**	**p = 0.01**
**HR-/HER2+**	**19**	**39.6 ± 6.0**	**84.3 ± 28.3**	**5.7 ± 1.9**	**p < 0.001**	**p > 0.05**	**p = 0.01**
**TN**	**16**	**38.4 ± 8.0**	**106.8 ± 13.4**	**7.9 ± 2.8**	**p < 0.001**	**p > 0.05**	**p < 0.01**

^a^Average ± standard error.

**Figure 2:**
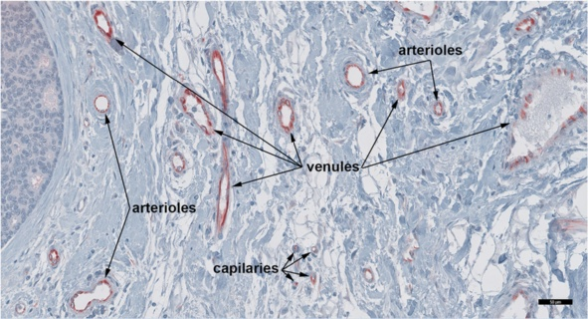
**Endothelial FSHR expression at the periphery of invasive breast cancer.** This picture illustrates that tumor FSHR+ blood microvessels are arranged in a hierarchical pattern of arterioles - capillaries - venules. Immunohistochemical analysis was performed on paraffin-embedded sections of human breast cancer tissues with the use of FSHR323 antibody, followed by a secondary peroxidase-coupled antibody visualized with the use of the red-brown peroxidase-reaction product of AEC. Scale bar represents 50 μm.

**Figure 3:**
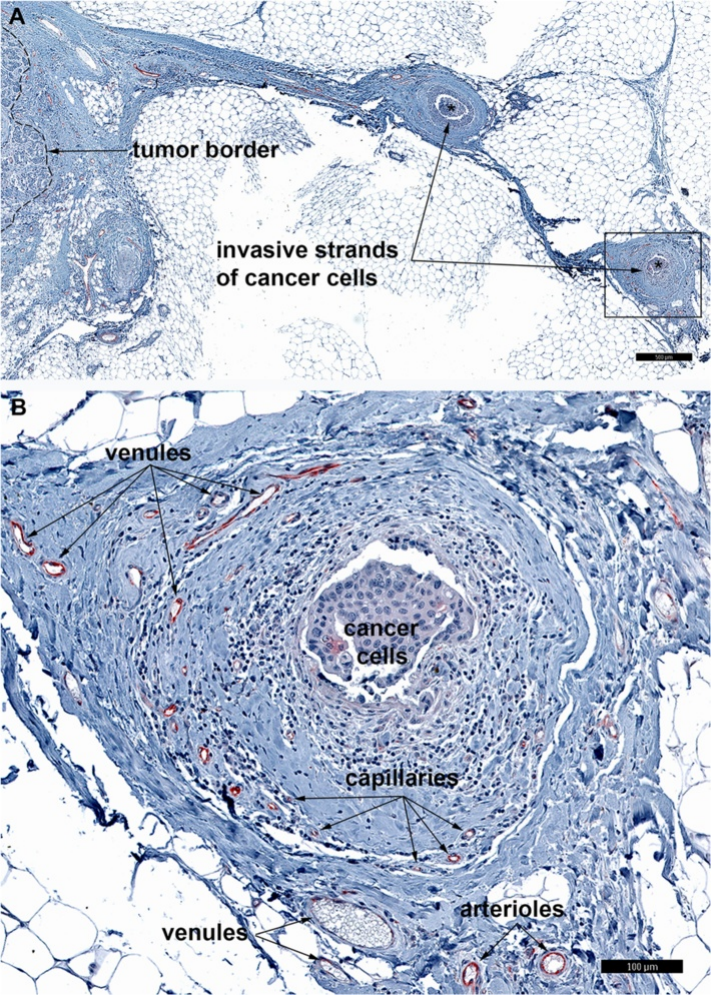
**FSHR expression in blood vessels associated with strands of invasive breast cancer cells. A)** Image of invasive strands of breast cancer cells (asterisk) at low magnification. **B)** Detail of an invasive strand of cancer cells located at 4 mm outside the tumor border (dashed line in **A**). Immunohistochemical analysis was performed as described in the legend of Figure 2. Scale bars: 500 μm **(A)** and 100 μm **(B)**.

The blood vessels that expressed FSHR were located in a layer that extended 2 mm into and 5 mm outside of the tumor. The percentage of blood vessels expressing FSHR reached a maximum of 100% at the demarcation line between the tumor and the normal tissue. FSHR+ blood vessels within these tumor zones (Figure 2) were similar in caliber and structural components to FSHR-negative breast vessels associated with the normal-appearing tissue located at a distance greater than 10 mm outside the tumors. Blood vessels associated with invasive tumor cell strands expressed also the endothelial FSHR (Figure 3). The total number of FSHR+ blood vessel we counted was 4,987. Approximately 62% of blood vessels expressing FSHR were capillaries (diameter ≤ 10 μm) and 25% were venules (10 μm < diameter ≤ 100 μm). Robust staining for FSHR was also found on small arterioles of ~ 25 μm in diameter (12%). Faint staining for FSHR was noticed on venules (100 μm < diameter ≤ 300 μm; 0.6%). All four molecular subtypes of breast cancer expressed almost similar densities of FSHR stained blood vessels (Table 1). Common among FSHR+ vessels, regardless of breast cancer type, were the high densities of arterioles and venules (6.4 ± 1.4 and 13.9 ± 2.1 vessels/mm^2^, respectively).

### Expression of angiogenic and lymphangiogenic markers at the periphery of invasive breast cancer

Positive staining for CD34 was observed in tumor core blood vessels, peritumoral vessels, and in blood vessels associated with normal breast tissue. Since endothelial cells seem to proliferate at periphery but not in the interior of the tumor [24], we quantified the CD34 staining associated with the tumor peripheral zone and with the normally-appearing breast tissue located at 1 cm from the tumor edge. In normal breast tissues the total number of CD34+ blood vessels we counted was 2,607 (4.6% arterioles, 84.5% capillaries, and 10.9% venules).

**Figure 4:**
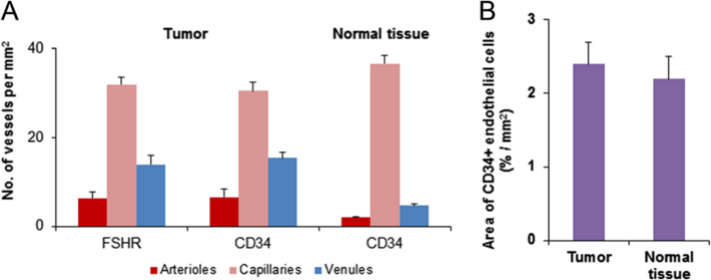
**FSHR expression and tumor peripheral vascular remodeling. A)** Tumor peripheral microvasculature contains 3-fold higher densities of arterioles and venules than that of normal breast tissue. **B)** The percentages of area occupied by CD34+ endothelial cells did not differ significantly among breast cancer peripheral tissue and normal breast tissue located at more than 10 mm from the tumor border. Values presented are means ± standard deviation (SD).

The densities of peritumoral CD34+ arterioles and CD34+ venules were similar to those that expressed FSHR, but, unexpectedly, they were 3-fold higher than that we noticed in normal breast tissue (Figure 4A). Insignificant differences were noticed between the percentages of area occupied by CD34+ endothelial cells/mm^2^ at the tumor periphery and in the normal breast tissue (Figure 4B). These results suggest changes in wall structure and diameter of blood vessels (vascular remodeling) rather than the increase of blood vessel number.

No specific staining for VEGFR2 protein was observed in tumor vessels, including the peripheral FSHR+ blood vessels. This negative result is not due to the anti-VEGFR2 antibody since with the same antibody, in identical conditions, we noticed a robust VEGFR2 staining associated with highly proliferative endothelial cells in human placenta (not shown).

By using the monoclonal antibody D2-40, the podoplanin-positive lymphatic vessels were identified in the peripheral tissue of all cases. The densities of D2-40+ lymphatic vessels ranged between 0.2 and 12.33 (Table 1). No significant differences were noticed between the breast cancer types (p > 0.05). Statistically significant positive correlations were found between the densities of peripheral tumor CD34+ blood vessels and peritumoral D2-40^+^ lymphatic vessels (p = 0.01).

### Correlations of FSHR expression with markers of angiogenesis and lymphangiogenesis

**Figure 5:**
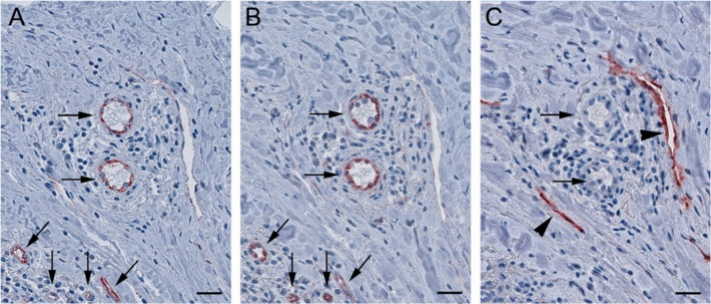
**Colocalization of FSHR with CD34 and D2-40 in tumor peripheral microvessels.** Immunohistochemical analysis was performed on three consecutive paraffin-embedded sections of human invasive breast cancer tissues by using the standard method with peroxidase. Blood vessels (arrows) stained for the endothelial marker CD34 **(A)** are FSHR+ **(B)**. By contrast, the D2-40-positive lymphatic endothelia (arrowheads) do not express FSHR **(C)**. Scale bars represent 20 μm.

As illustrated in Figure 5A and B, the vessels which are stained for CD34 are also FSHR+. In contrast, D2-40^+^ lymphatic vessels (Figure 5C) do not express FSHR. The densities of FSHR+ vessels, peritumoral CD34+ blood vessels, and D2-40^+^ lymphatic vessels for all molecular subtype of breast cancer are shown in Table 1. Statistically significant correlations were found between the densities of FSHR+ vessels and peritumoral CD34+ blood vessels. No correlation was observed between the density of FSHR+ vessels and peritumoral D2-40^+^ lymphatic vessels’ density (p > 0.05) (Table 1).

### Correlation of FSHR expression with clinicopathological data

We have finally compared the FSHR expression with the tumor grade and size, age of patients, and with the receptor status of ER, PR, and HER2. Significant positive correlation was found between the density of peritumoral FSHR+ blood vessel and tumor size (Table 2). We have also noticed a tendency (not reaching statistical significance) towards an overall decrease in the extent of the vascular bed in elder patients (age > 50 years) as compared to younger patients. No correlations were found between the density of FSHR+ vessels and either the expression of ER, PR, and HER2, or tumor grade (Table 2).

**Table 2:** **Correlation of FSHR expression with clinicopathological data**

**Clinicopathological data**	**No of cases**	**r- value** ^**a**^	**p - value**
**Age**	**83**	**- 0.2149**	**p = 0.051**
**Tumor size**	**43**	**0.38758**	**p = 0.01**
**Grade of tumor**	**43**	**0.08785**	**p > 0.05**
**ER status**	**23**	**0.16477**	**p > 0.05**
**PR status**	**23**	**0.16477**	**p > 0.05**
**HER2 status**	**26**	**- 0.1400**	**p > 0.05**

^a^Pearson correlation coefficient.

## Discussion

In agreement with previous studies [21,22], our present results indicate the presence of specific endothelial cell receptors for FSH at the periphery of breast tumors and add new data on FSHR expression in blood vessels associated with the invasive cancer cell strands. We show for the first time that, in contrast to tumor core vasculature which is disorganized and tortuous [28,29], the peripheral tumor FSHR+ blood microvessels are arranged in a hierarchical pattern: arterioles - capillaries - venules.

In normal tissues, the vascular networks are topologically and structurally heterogeneous and include long and short blood flow pathways. While, long blood flow pathways (feeding arterioles) supply in oxygen and nutriments more remote tissue regions, the short flow pathways (arterio-venous shunts) supply tissues adjacent to feeding vessels [30]. To avoid competition with normal cells, cancer cells must modify these vascular networks. The abnormal high densities of arterioles and venules that we have observed at the tumor periphery suggest that breast cancer cells can switch the blood circulation from long to short flow pathways. Since the percentage of blood microvessels expressing FSHR reached a maximum of 100% at the demarcation line between the tumor and the normal tissue we expect that the endothelial FSHR play an important role in vascular remodeling and generation of short low-resistance, high-flow pathways of blood at tumor periphery. Therefore, blocking the formation of functional shunts by inhibiting endothelial FSH/FSHR signaling may be a new strategy in cancer therapy.

In this study we analyzed FSHR expression in patients that had small lymph node-negative invasive tumors. For these early-stage invasive cancers, surgery is a standard. Breast-conserving surgery is often the appropriate therapy, although mastectomy is also an option. Clinical trials have shown that breast-conserving surgery plus radiation therapy is as effective as mastectomy. However, the incidence of recurrent tumor in the same breast is 8.8-fold higher after 20 years among patients treated with breast-conserving surgery than that noticed in the radical-mastectomy group [31]. The latter results indicate that cancer cells were missed in some cases and, therefore, objective determination of resection margins is required to improve resection of the invasive strands. Our present results show that, in small lymph node-negative invasive tumors, the blood vessels that expressed FSH receptor were located at the tumor periphery in a layer that extended 2 mm into and 5 mm outside of the tumor. We have also noticed that blood vessels associated with invasive tumor strands expressed also the endothelial FSHR. These findings indicate that resection of tumor margin guided by an FSHR-specific signal [32] may prove to be sufficient to completely remove the tumor, irrespective of breast cancer molecular subtype. (All four clinical treatment groups of breast cancer we have analyzed (HR+/HER2- tumors, HR-/HER2+ tumors, HR+/HER2+ tumors, and TN breast tumors) expressed similar densities of tumor peripheral FSHR+ blood vessels).

Drug therapy is also recommended for treatment of patients with invasive breast cancer. This may include hormone therapy, HER2 targeted drugs, and chemotherapy. Due to tumor heterogeneity, combinations of these treatments are often recommended. Depriving a tumor from its oxygen and nutrients by preventing the formation of new vessels (angiogenesis) is also used as a treatment modality in oncology [15]. However, the clinical impact from antiangiogenic treatment alone or in combination with standard chemotherapeutic regimens has been relatively small till today [33].

Disruption of vessels already present in the core of tumors has been also envisaged [16-18] but the efficacy of this anti-vascular therapy is substantially compromised by the inability of drugs to completely kill tumor cells located at the periphery of the tumor mass [19]. Therefore, treatments targeting the tumor peripheral vessels, as those expressing FSHR, may be logically more likely to produce a cure compared to those targeting pathologically defective tumor core vessels. A recent study confirmed our hypothesis that interruption of the endothelial FSH/FSHR signaling on colon tumor-associated blood vessels induces reduction of tumor vasculature, radiological stabilization and carcinoembryonic antigen stabilization during 1 year [34].

## Conclusions

Our results indicate, for the first time, changes in wall structure and diameter of tumor peripheral blood vessels (vascular remodeling), a cellular process in which the endothelial FSH/FSHR signaling should play an important role. From a clinical standpoint, this novel observation adds value in augmenting the current receptor-based biomarkers for therapy of invasive breast cancer.

## Abbreviations

AEC: Amino-ethyl carbazole
ER: Estrogen receptor
FSH: Follicle-stimulating hormone
FSHR: Follicle-stimulating hormone receptor
HER2: Human epidermal growth factor receptor 2
HR: Hormone receptor
TN: Triple negative
VEGFR2: Vascular endothelial growth factor receptor 2
